# Impact of the COVID-19 Pandemic on the Lifestyle and Psychosocial Behavior of Patients with Inflammatory Bowel Diseases: A Narrative Review

**DOI:** 10.3390/healthcare11192642

**Published:** 2023-09-28

**Authors:** Yu Nishida, Shuhei Hosomi, Yumie Kobayashi, Rieko Nakata, Masaki Ominami, Yuji Nadatani, Shusei Fukunaga, Koji Otani, Fumio Tanaka, Yasuaki Nagami, Koichi Taira, Noriko Kamata, Yasuhiro Fujiwara

**Affiliations:** Department of Gastroenterology, Graduate School of Medicine, Osaka Metropolitan University Osaka, Osaka 530-0001, Japan

**Keywords:** inflammatory bowel disease, coronavirus, pandemic, lifestyle, physical activity, sleep time, drug adherence

## Abstract

The coronavirus disease (COVID-19) pandemic has had a considerable impact on the global healthcare system and potentially the clinical course of patients with inflammatory bowel disease (IBD). Although IBD is a chronic disease, its therapy (except steroid therapy) does not increase the risk of contracting or aggravating COVID-19. However, the clinical course of patients is significantly influenced by environmental factors. Social restrictions due to the pandemic or the fear of contracting the virus have influenced lifestyle and psychosocial behaviors that may worsen the clinical course of patients with IBD. This narrative literature review summarizes the current evidence on the impact of the COVID-19 pandemic on the lifestyle and psychosocial behaviors of patients with IBD. The COVID-19 pandemic negatively affected the lifestyle and psychosocial behaviors of patients with IBD. Furthermore, patients with IBD failed to maintain medication adherence, thus affecting the clinical course of their condition.

## 1. Introduction

The coronavirus disease (COVID-19) pandemic, caused by severe acute respiratory syndrome coronavirus 2 (SARS-CoV-2), is a public health emergency affecting the global healthcare system, and this may change the clinical course of the patients with inflammatory bowel disease (IBD). COVID-19 was first detected in Wuhan, China, in late 2019, and it rapidly spread worldwide [1]. This viral disease is the cause of respiratory infections with mild-to-moderate pneumonia manifestations [1]. SARS-CoV-2 infects the host by binding to the angiotensin-converting enzyme II (ACE2) receptor, which is highly expressed in the intestinal tract organs [2]. In total, 12.5% of patients with COVID-19 have been reported to experience diarrhea, and viral RNA has been detected in feces [3]. There is a close relationship between COVID-19 and gastrointestinal symptoms.

When COVID-19 was declared a global health emergency by the World Health Organization, many countries, particularly the highly affected countries, implemented lockdowns as a measure to prevent the spread of the virus. Various restrictions were implemented in most countries to prevent infection spread. Common strategies included social distancing and lockdowns, resulting in considerable lifestyle changes, affecting physical activity, dietary habits, sleep patterns, childcare, work burden, and medical service access [4,5,6]. In addition to these social restrictions, the pandemic led to widespread fear and anxiety about the infection and its potential complications, thus contributing to a negative psychological impact [7]. The pandemic has impacted mental health, resulting in social isolation, loneliness, and financial strain, as well as uncertainty for the future and anxiety/fear about contracting the virus. Changes in physical activity, sleep pattern, eating behavior, time use, childcare, and mental health are unprecedented, and the COVID-19 crisis inevitably reinforced the link between lifestyle behaviors and depression worldwide [8]. IBD is a heterogeneous group of inflammatory gastrointestinal tract disorders with a chronic or recurrent clinical course. This disease is characterized by phases of exacerbation and remission. Several factors have been identified as potential risk factors for a poor outcome during COVID-19, including active disease, combined immunosuppression, corticosteroid therapy, advanced age, cardiovascular disease, diabetes, and hypertension [9]. Besides corticosteroid use, IBD is not a major risk factor for SARS-CoV-2 infection or its exacerbation after infection [10,11,12]. Disease activity can be influenced by environmental factors, including sleep, mental stress, diet, smoking, and medication adherence [13,14,15,16,17]. However, social restrictions may potentially exacerbate IBD or alter its clinical course, affecting lifestyle and psychosocial behaviors, or fear of contracting COVID-19.

Hence, evaluating lifestyle and psychosocial behavior changes in patients during the pandemic is crucial for choosing appropriate therapies and providing appropriate lifestyle education. This review provides a prevalence overview of lifestyle changes to understand how to manage patients with IBD during the pandemic. This narrative literature review covers the period from December 2019 to July 2023.

## 2. Sleep Changes during the Pandemic in Patients with IBD

### 2.1. Association between COVID-19 and Sleep Status

The COVID-19 pandemic negatively impacted sleep patterns in the general population [18]. Furthermore, sleep disturbances were reported to be highly prevalent in patients with IBD, increasing the risk of relapse and chronic fatigue [19]. Shorter sleep hours were reported to be a risk factor for relapse in patients with IBD [13,14,15]. However, studies focusing on the sleep status of patients with IBD during the pandemic remain scarce. Koletzko et al. observed a significant increase in self-reported sleep duration during the pandemic compared with that before the pandemic. Sleep duration changes during the pandemic significantly differed between age groups, with an increase in children and young adults and a decrease in elderly people [20]. Similarly, Nishida et al. reported increased sleep duration during the pandemic [21]. Some studies reported decreased sleep quality [20,22], while others reported no negative impacts [23,24] in patients with IBD during the pandemic. However, almost half the IBD patients reported insomnia during the pandemic [25]. Furthermore, sleep disturbances have been associated with IBD exacerbation in patients with ulcerative colitis (UC) during the pandemic [26]. It is difficult to objectively evaluate sleep time and sleep quality, which may be the reason for the lack of consistency.

### 2.2. Improving Sleep Status during the COVID-19 Pandemic

Sleep disturbance can be addressed through interventions by physicians or patients. However, no study has evaluated whether improved sleep conditions improve the clinical course of IBD or its objective measures during the COVID-19 pandemic. Further studies are required to evaluate the importance of improving sleep quality. However, poor sleep quality is reported to be independently associated with fatigue [27], and improving sleep conditions may reduce fatigue and improve quality of life in patients with IBD. Sleep disturbance assessments may be important in disease monitoring and optimizing medical management to prevent disease relapse [28]. COVID-19 may have led to a decrease in sleep quality. Patients who experienced sleep disturbances during the pandemic should be referred to sleep specialists for medication; this approach may help reduce disease activity in patients with IBD.

## 3. Physical Activity Changes in Patients with IBD

### 3.1. Impact of the COVID-19 Pandemic on Physical Activity in Patients with IBD

Physical activity levels can influence the clinical course of patients with IBD [29]. Jones et al. reported that higher physical exercise levels were a preventive factor against symptomatic relapse among patients in remission [29]. Physical activity recommendations advocate maintaining an active lifestyle of endurance and resistance exercise [30].

The COVID-19 pandemic may cause behavioral changes due to government restrictions or fear of contracting infections. Di Renzo et al. reported that individuals who exercised before the pandemic occasionally exercised more at home during the pandemic; however, individuals who had no habit of playing sports remained inactive during the lockdown [31]. Most studies on the impact of the COVID-19 pandemic on physical activity behavior among the general population have revealed that physical activity levels decreased during the COVID-19 lockdown, with an increase in sedentary behavior [32,33,34,35,36]. However, COVID-19 precautions should not prevent people from being physically active.

Given the correlation between physical activity and the clinical progression of IBD reported in previous studies, monitoring physical activity level evolution among patients with IBD during the pandemic is essential. Although reduced physical activity in patients with IBD during the pandemic was reported to be a risk factor for worsening self-evaluations of the disease [37], patients with IBD stayed at home more frequently than non-IBD patients and left the house less frequently than before the pandemic [38]. The study reported that this finding may be due to a greater fear of SARS-CoV-2 infection in patients with IBD than in those without, especially in individuals using immunosuppressive drugs [38]. Reinsch et al. reported a similar trend among pediatric patients with IBD who left home less frequently than they did before the COVID-19 pandemic [39]. Nishida et al. reported that exercise duration decreased during the COVID-19 lockdown compared to during the pre-lockdown period, especially in non-elderly patients [21].

### 3.2. Improving Sleep Status during the COVID-19 Pandemic

Most patients recognize the importance of exercise; however, lockdown measures, such as strict social distancing, school closure, and mass gathering restrictions, have dramatically altered traditional lifestyles, including regular exercise. Although exercise recommendations already exist [40], there is a greater need for support to help patients use these guidelines effectively and understand inactivity implications. Health literacy encourages regular exercise [41]. Patient guidance is important because physical activity in IBD patients, as in the general population, decreases during an epidemic, and decreased physical activity is reportedly associated with worsening IBD symptoms. During the pandemic, improved health literacy could assist in maintaining or establishing regular exercise habits in the daily life of patients. Therefore, physician guidance and social support are essential for maintaining physical activity.

## 4. Medication Adherence in Patients with IBD during the Pandemic

### 4.1. Association between Medication Adherence and Clinical Course in Patients with IBD

In patients with IBD, medication nonadherence has been reported to be associated with increased disease activity [17,42], poor quality of life [43,44], loss of response to antitumor necrosis factor (TNF) therapy [45], increased healthcare expenditure, and higher morbidity rates [46]. Notably, due to anti-TNF therapy discontinuation, 55% of the patients with Crohn’s disease or UC relapsed after 32 and 18 months, respectively [47].

### 4.2. Impact of the COVID-19 Pandemic on Medication Adherence in Patients with IBD

Patients with IBD do not have a higher risk of developing COVID-19 or worse virulence once infected, except those using corticosteroid therapy [48]. Therefore, patients with IBD are recommended to continue their treatment to prevent exacerbations [49,50,51]. However, this situation may cause inappropriate behaviors during the pandemic, including medication discontinuation, due to the misunderstanding that medication should not be used due to its immunosuppressive effects [52]. Furthermore, continuing medication during the pandemic may have been difficult for patients due to barriers to hospital visits or their irregular lifestyles. The European Crohn’s and Colitis Organization (ECCO) guidelines recommend considering subcutaneous drugs to minimize hospital visits for IBD flares [53]. However, Lindhagen et. al. reported that the proportion of patients who changed their medical treatment in the first year of the pandemic did not change, compared with the proportion in the pre-pandemic year [54].

There are many drug adherence rate reports of patients with IBD during the pandemic period, with results varying by geographic location, term, and medication categories. Review articles have reported that drug discontinuation rates ranged from 3.73% to 28%, and infusion delay rates ranged from 1.3% to 69.6% [55]. Several studies conducted at the beginning of the pandemic reported that nonadherence rates to IBD therapies, including biologics, during the COVID-19 pandemic, were relatively high, especially in facilities that halted biologics during this period [56,57]. Kahn et al. [58] reported that the proportion of patients with IBD receiving an infusion of biologic therapy within 10 weeks of the prior infusion decreased from 84.6% to 73.6% between 2019 and 2020, with a persistent drop in the weekly infusion number from late March 2020. Bezzio et al. reported that 30.7% of the patients had delayed or discontinued IBD therapy [59]. Chen et al. reported that 27.7% (107/386) of patients discontinued their medication during the COVID-19 pandemic for reasons such as social restrictions and hospital regulations to prevent infection spread, contracting COVID-19, or adverse drug effects [60]. Medication adherence rates vary by medicine category. Harris et al. reported that oral steroids are the most frequently discontinued drugs. A total of 41% (12/29) and 11.2% (33/294) of patients stopped prednisolone and immunomodulators, respectively. However, 4.1% (13/310) of patients discontinued biological treatments, with three of them discontinuing their biological therapy by themselves [61]. However, other studies have reported low treatment discontinuation rates during the pandemic. Barnes et al. found that only 5% of patients reported self-initiated missed doses or dose reductions of IBD medications directly attributed to the COVID-19 pandemic. Furthermore, they noted that COVID-19 prevalence during the survey was low, and that patients in areas with a higher prevalence may exhibit greater nonadherence rates [62]. In a survey conducted with the support of the European Federation of Crohn’s and Ulcerative Colitis Association, 4% of the respondents stopped using IBD medication during the pandemic on their own [52]. Nakase et al. reported that only 0.6% of patients discontinued therapy, 1.9% used medications at reduced doses, and 97.5% used medications as directed [63]. These results suggest that good adherence is possible despite the ongoing disruptions caused by the pandemic. As expected, patients who discontinued treatment were more likely to relapse [64]. Therefore, it is important to identify methods by which to prevent treatment discontinuation.

Medication nonadherence has been reported to be caused by multiple factors associated with patients (fear of adverse effects or immunosuppression), social restrictions, facility closure, and physician factors (fear of infection or adverse effects) [56,57,65]. When infection rate is high, medication adherence is assumed to decline because of hospitals being closed or people not being able to visit hospitals; however, in cases where an epidemic has subsided, adherence is assumed to decline because of worsening mental status or other factors. Nakase et al. reported that the most common reasons for discontinuing medications were “stable abdominal symptoms” (38.4%), “instructions from the attending physicians” (31.5%), and “a higher risk of contracting infection due to medication” (21.9%) [63]. Although physicians should communicate with patients to help them understand the necessity of continuing medication therapy, only 7% of the patients reported discussing their medication with their medical staff during the pandemic [66]. Many patients with IBD lack comprehensive information on the association between COVID-19 and IBD. Sufficient communication may be difficult in a pandemic due to the difficulty of conducting face-to-face examinations or short medical visits. These barriers may cause poor adherence due to a lack of understanding about continuing treatment or misunderstanding that treatments would negatively affect COVID-19. Providing appropriate COVID-19 information to patients with IBD may reduce pandemic-related anxiety [64] and improve drug adherence. Telemedicine could be a valid method for communicating with patients; however, further patient efforts are needed in order to improve adherence and increase its use in daily practice [67]. Thus, physicians should communicate with patients and provide appropriate health-related guidance to maintain treatment. The selected study findings on medication adherence in patients with IBD during the pandemic are summarized in Table 1.

## 5. Impact of the COVID-19 Pandemic on Disease Activity in Patients with IBD

### 5.1. Assessment of Disease Activity in Patients with IBD

The COVID-19 pandemic affected multiple factors influencing the clinical course of IBD, including lifestyle behaviors, psychological status, and drug adherence. Several outcomes can be considered regarding the impact on disease activity, including patient-reported outcome measures, flare-ups, fecal calprotectin, hospitalization rates, or emergent surgeries. Several studies have evaluated these outcomes to analyze the impact of the COVID-19 pandemic on disease activity among patients with IBD.

### 5.2. Patient-Reported Outcomes

Yu et al. reported that 53.92% of the patients thought their symptoms did not change during the pandemic, 24.51% thought their disease condition had improved, and 21.57% believed their symptoms had worsened during the pandemic. Using patient-reported outcomes, Nishida et al. reported that gastrointestinal symptoms worsened in 34.0% and 46.2% of patients with UC and Crohn’s disease during the pandemic, respectively [26]. Goodday et al. reported that the severity of self-reported Crohn’s disease was significantly different between the pre-pandemic and pandemic periods: 17% of previously inactive patients became active during the pandemic period [68].

In an anonymous questionnaire survey, Rizzello et al. reported that 38.4% of the patients on biological therapy had worsened IBD symptoms during the national lockdown period. Among these, 84.5% of patients with worsened symptoms stopped or prolonged their biological therapy [69].

### 5.3. Fecal Calprotectin

In an objective outcome study, Lindhagen et al. reported no difference in the proportion of patients with median fecal calprotectin levels between the first pandemic year and pre-pandemic year. Furthermore, there were no proportion differences in patients who changed their medical treatment during these periods [54]. Objective evaluations of disease activity include pathological and endoscopic evaluations; however, to the best of our knowledge, no study has evaluated disease activity changes determined using endoscopic or pathological examinations during the pandemic. This may be because the number of endoscopy procedures decreased during the pandemic [70]. Early in the pandemic, most guidelines recommended postponing routine or elective endoscopies in patients with IBD [71,72]. It may have been difficult to design a study that involved endoscopic evaluation, as this carries the risk of infection spread.

### 5.4. Hospital Admission Rate

Martinelli et al. reported a significant reduction in IBD flare-related hospital admissions in a pediatric IBD study; however, this was not due to fewer relapses requiring hospitalization but because patients requiring hospitalization were adequately treated [73].

### 5.5. Outlook for the Future

There is controversy regarding whether COVID-19 affects IBD activity. This is because the prevalence of infection, countermeasures, and completeness of countermeasures vary by region and time of the study. In addition, clinical course evaluations vary, which may be the reason for the discrepancy in results.

Face-to-face medical examinations were more difficult during the pandemic due to social restrictions. Telemedicine, e-mail, and mobile communication applications were recommended to understand patient status, detect those requiring urgent care, and identify individuals who could postpone their medical examinations [7,74].

## 6. Influence of the COVID-19 Pandemic on Psychological Status in Patients with IBD

### 6.1. Mental Health in Patients with IBD in COVID-19 Pandemic Era

The COVID-19 pandemic affected mental health conditions owing to social restrictions and fear of infection, including depression and anxiety [75]. The isolation measures created to prevent COVID-19 contraction inflicts stress upon these patients and may cause increased depressive symptoms. Patients with IBD, particularly those with active disease, are more likely to develop anxiety or depression than healthy people, even during the non-pandemic era [76,77]. Furthermore, these patients need regular hospital visits to prevent recurrence, and the mental epidemic burden on them is considered greater than that in the general population. This is because patients with IBD may fear contracting COVID-19 during hospital visits or worry about the difficulty of visiting the hospital due to pandemic restrictions. Several studies have reported negative effects of the pandemic on the psychological status of patients with IBD. Trindade et al. reported that approximately half of their study population presented with moderate (37.10%) to severe (14.50%) anxiety [78]. Feitosa et al. reported that patients developed depressed mood (80.2%), anxiety and fear of death (58.2%), insomnia (51.4%), daily activity disturbances (48%), sexual dysfunction (46.2%), and productivity disorders (44%) [25]. Patients were concerned about infection risks due to hospital visits and that their illness or IBD medication may increase their COVID-19 susceptibility [38,63,78,79]. D’Amico et al.’s study revealed that 85% of patients with IBD were anxious about the risk of developing COVID-19, and 64% believed that immunotherapy was associated with a greater infection risk [52].

### 6.2. Impact of the COVID-19 Pandemic on Psychological Status

Only a few studies have evaluated the impact of the pandemic on the psychosocial status of patients with IBD, comparing the pre-pandemic and pandemic periods. This may be because studies that compare mental states between these periods are difficult to design. Nishida et al. revealed that the stress of being unable to exercise and staying indoors increased significantly during the lockdown compared with the period before the pandemic. Additionally, increased stress due to the COVID-19 pandemic was identified as an independent UC exacerbation risk factor [21,26]. Harris et al. reported that lockdowns increased the stress levels of patients, and this stress persisted after the restrictions were eased. Additionally, they reported that patients felt even more stress in the subsequent COVID-19 wave [61]. Goodday et al. reported that the proportion of respondents who experienced stress increased. In the period before the COVID-19 pandemic, 31% (73/236) of the patients felt stressed; whereas during the COVID-19 pandemic era, this number increased to 52% (122/236) among patients with Crohn’s disease [68]. However, Luber et al. reported that 22.5% (61/271), 18% (49/271), and 14% (39/271) of the patients had moderate-to-severe depression, moderate-to-severe anxiety, or both, respectively. They concluded that these rates were similar to those of the pre-pandemic period and the general population [80]. However, no studies have reported the psychological effects of COVID-19 in patients with IBD, and this should be addressed in the future.

### 6.3. Factors Associated with Depression and Anxiety in Patients with IBD during the Pandemic

Several COVID-19 factors have been reported to be associated with depression and anxiety in patients with IBD during the pandemic, including their general condition, self-isolation, employment status, fear of visiting the hospital, and difficulty accessing information [78,80,81]. Table 2 summarizes the findings on the influence of the COVID-19 pandemic on the psychological status of patients with IBD during the pandemic.

### 6.4. Comparing the Psychological Impact of the Pandemic at the Beginning and at the End of the Pandemic

Sempere et al. [82] reported that after lockdown, anxiety and depression improved markedly. During lockdown, comorbidity, active IBD, use of biologics, and living alone or with one person were identified as risk factors for depression symptom. This suggested that patients may not have received proper care from the healthcare system due to the lockdown or they may have lacked social support during the lockdown. However, after lockdown, factors associated with depression were those commonly found in patients with IBD, such as the disease activity of IBD or previous mood and/or anxiety disorders. This suggests that many participants’ conditions improved toward the end of the lockdown. It is believed that the impact of the pandemic on psychosocial behavior will not be as great at the end of the pandemic compared with the early stages.

Providing appropriate therapy to avoid disease flares as well as sufficient and appropriate information regarding COVID-19 is important to protect patients with IBD from mental disorders during the pandemic. Easy access to information regarding COVID-19 risks may help reduce the psychological burden.

In the future, even as COVID-19 becomes a common infectious disease, easily obtaining appropriate information related to this disease may help reduce mental burden. Additionally, this could provide a necessary lesson for future waves of infections, variants, and epidemics.

## 7. Conclusions

### 7.1. Lifestyle and Psychosocial Behavior of Patients with IBD during the COVID-19 Pandemic

The COVID-19 pandemic negatively affected the lifestyle and drug adherence of patients, particularly at the beginning of the pandemic. The lifestyles of many patients were disturbed. Although the association between infection prevalence and physical activity or psychosocial behavior is inconsistent, improved health literacy may assist in maintaining or establishing regular exercise habits in the daily life of patients. Therefore, physician guidance and social support are essential for maintaining physical activity. Furthermore, patients with IBD failed to maintain medication adherence during the pandemic, affecting their clinical course. Patients should maintain a positive emotional status, continue medication as directed, retain the medication regimen, have adequate rest, and communicate closely with healthcare providers during a pandemic.

### 7.2. Future Directions

Although COVID-19 will become a common infectious disease and restrictions will ease, including the COVID-19 lockdown, the emergence of novel viruses or bacteria and new outbreaks may affect human lives and the healthcare system. Considering the concerns and vulnerabilities of patients with IBD, lessons learned from this pandemic will be important for future outbreaks. If a new wave or a similar pandemic occurs, caution when postponing IBD care is essential in order to prevent exacerbations among these patients. These studies demonstrate that patients can quickly adapt to new situations when clear and consistent communication is maintained. Noninvasive disease assessment methods, telemedicine, and subcutaneous treatment recommendations to decrease hospital visits will be useful for this purpose. Based on the lessons learned from this pandemic, healthcare providers must communicate sufficiently and effectively with patients to prevent IBD exacerbation.

## Figures and Tables

**Table 1:** Summary of medication adherence in patients with IBD during the pandemic.

Author	Summary of Findings	References
Tian W.N.	A questionnaire survey examined 239 patients in China. During the COVID-19 pandemic, 21.76% of the patients changed their medication, while 31.14% of the patients reported that they could not continue medication. The main reason was that many hospitals were unable to open and treat patients who had not been diagnosed with COVID-19.	[56]
An P.	There were 318 patients with IBD registered in a prospective database in Wuhan, China, between 1 January 2000, and 8 December 2019. On 3 January 2020, infliximab infusion and immunosuppressive treatment were discontinued for all patients according to the Chinese Society of Gastroenterology national guidelines.	[57]
Khan N.	A nationwide retrospective cohort study using US National Veterans Affairs health care system data from the Veterans Affairs Informatics and Computing Infrastructure was conducted. This study included 2510 and 2516 patients with IBD in 2019 and 2020, respectively. The proportion of IBD cases receiving an infusion within 10 weeks of the prior infusion with biologics decreased from 84.6% to 73.6% between 2019 and 2020, with a persistent drop in the weekly number of infusions since late March 2020.	[58]
Bezzio C.	A prospective, multicenter, nationwide case-control study was conducted in Italy, evaluating 219 patients with IBD who had a SARS-CoV-2 infection diagnosis and 219 with IBD without infection. Notably, 30.7% (125/406) of the patients delayed or discontinued their IBD therapy. IBD and SARS-CoV-2-infected patients had significantly higher delay or discontinuation rates than those without viral infection (*p* < 0001).	[59]
Chen J.	A questionnaire survey examined 386 IBD patients who were registered in the electronic medical record system at the IBD Center of the Sixth Affiliated Hospital of Sun Yat-sen University in China from 23 January 2020 to 23 April 2020. A total of 27.7% (107/386) patients discontinued their medication during the COVID-19 pandemic because it influenced social restrictions and hospital regulations to prevent infection spread, concerns of contracting COVID-19, or adverse drug effects.	[60]
Harris R.J.	A questionnaire survey examining 85 patients with IBD in the UK from 16 June to 21 August 2020. Oral steroids were the most frequently discontinued drug, with 41% (12/29) and 11.2% (33/294) patients stopping prednisolone and immunomodulators, respectively. However, 4.1% (13/310) of patients discontinued biological treatments, with three of these patients discontinuing their biological therapy by their own choice.	[61]
Barnes A.	An anonymous online survey examined 262 patients with IBD from May 2020 to July 2020. Only 5% of the patients reported self-initiated missed doses or dose reduction of their IBD medications that was directly attributed to the COVID-19 pandemic.	[62]
Nakase H.	A questionnaire survey examined 3032 patients with IBD in Japan between March 2020 and June 2021. Only 0.6% of the patients discontinued therapy and 1.9% took medication in reduced doses. Furthermore, 97.5% of the patients used the medicine as directed. Stable gastrointestinal symptoms (38.4%) were the most common reason for patients to reduce or discontinue their medication, followed by orders of the physician (31.5%) and a high risk of drug-induced infection (21.9%).	[63]
Peng Y.L.	A prospective multicenter study examined 469 patients with IBD in Taiwan between July 2021 and December 2021. Notably, 7.42% of the patients self-discontinued their IBD drugs. The most discontinued drugs were immunomodulators (2.54%), mesalazine (2.12%), and steroids (1.23%). Of those who discontinued treatment, 34.28% reported worsening IBD symptoms.	[64]
D’Amico F.	A questionnaire survey conducted with the support of the European Federation of Crohn’s and Ulcerative Colitis Associations examined 3815 patients with IBD in 51 countries between 30 March and 16 April 2020. Of these patients, 81% were unwilling to discontinue their IBD medications during the pandemic, and 96% did not discontinue their IBD medications using their self-judgment.	[52]

COVID-19, coronavirus disease; IBD, inflammatory bowel disease.

**Table 2:** Summary of the influence of the COVID-19 pandemic in patients with IBD during the pandemic.

Author	Summary of Findings	References
Trindade I.A.	An online questionnaire survey examined 128 Portuguese individuals in April 2020. Normal, mild, moderate, and severe anxiety levels were 29.80%, 18.50%, 37.10%, and 14.50%, respectively. Furthermore, normal, mild, moderate, and severe depression levels were 51.60%, 27.40%, 16.10%, and 4.8%, respectively.	[78]
Feitosa M.R.	A questionnaire survey using telephonic consultations examined 179 patients in Brazil. Patients developed depressed moods (80.2%), anxiety and fear of death (58.2%), insomnia (51.4%), daily activity disturbances (48%), sexual dysfunction (46.2%), and productivity disorders (44%). No significant differences were noted between active and inactive diseases for patients with Crohn’s disease and ulcerative colitis.	[25]
Grunert P.C.	An anonymous questionnaire survey examining 415 patients with IBD and 116 control participants in Germany between 2 and 17 April 2020. Compared to control participants, patients with IBD were more afraid of contracting COVID-19 (*p* = 0.009) and were more concerned about the negative drug effects on COVID-19.	[38]
Nakase H.	A questionnaire survey examining 3032 patients with IBD in Japan between March 2020 and June 2021. Participants demonstrated an increased trend of anxiety a month after the number of infected people per population increased. Women, homemakers, hospital arrival time from home, commuting to the hospital by train, and IBD medication were identified as significant anxiety factors.	[63]
D’Amico F.	A questionnaire survey examining 3815 patients with IBD in 51 countries between 30 March and 16 April 2020. Findings revealed that 85% of the patients feared contracting COVID-19, 87% of the patients were afraid of traveling, and 74% of the patients were afraid of attending their hospital for follow-up consultations.	[52]
Nishida Y.	A questionnaire survey examining 451 patients with IBD in Japan from 16 June to 21 August 2020. Increased stress due to the COVID-19 pandemic was identified as an independent UC exacerbation risk factor (OR, 6.06; 95% CI, 1.79–20.50; *p* < 0.01)	[26]
Nishida Y.	A questionnaire survey examining 451 patients with IBD in Japan from 16 June to 21 August 2020. Stress associated with being unable to exercise and having to stay indoors increased significantly during the lockdown compared to that before.	[21]
Harris R.J.	A questionnaire survey examining 685 patients with IBD in the UK from 16 June to 21 August 2020. During this period, 14.9% of the patients had anxiety or depression as comorbidities. Lockdowns increased patient stress levels, which persisted after restrictions eased. Furthermore, patients experienced more stress with the subsequent COVID-19 wave.	[61]
Goodday S.M.	An anonymous survey was distributed to 243 patients with Crohn’s disease in England between 30 April and 26 June 2020. Among patients with Crohn’s disease, 31% (73/236) felt stressed during the pre-COVID-19 pandemic era, while 52% (122/236) felt stressed during the pandemic. Furthermore, 17% of the patients with Crohn’s disease transitioned from an inactive to an active phase during the pandemic. Increased stress was the most common reason for these symptom exacerbations.	[68]
Luber R.P.	A questionnaire survey was distributed to 271 patients with IBD in the United Kingdom in June 2020. There were 22.5% (61/271) patients with moderate-to-severe depression, 18% (49/271) had moderate-to-severe anxiety, and 14% (39/271) had both. This study concluded that these rates were similar to those in the pre-pandemic period and general population. Several factors are reported to be associated with depression or anxiety in patients with IBD during the pandemic era, including fatigue, patient suspicion of a flare, using a psychological intervention, and difficulty accessing risk information.	[80]

COVID-19, coronavirus disease; CI, confidence interval; IBD, inflammatory bowel disease; OR, odds ratio.

## Data Availability

All the data are included in this manuscript.
